# Crystal-field splitting strength of U-6*d* orbitals in NaUO_3_, KUO_3_ and RbUO_3_

**DOI:** 10.1107/S1600577525005156

**Published:** 2025-07-30

**Authors:** Simon Orlat, Igor Prozheev, Ine Arts, Gregory Leinders, Elena Bazarkina, Kristina Kvashnina, Filip Tuomisto, Philippe Martin, Philippe Moisy, René Bes

**Affiliations:** a Department of Physics University of Helsinki PO Box 64 FI-00014Helsinki Finland; b Helsinki Institute of Physics PO Box 64 FI-00014Helsinki Finland; c CEA/DES/ISEC/DMRC University of Montpellier Bagnols sur Cèze France; d EMAT, Department of Physics University of Antwerp Groenenborgerlaan 171 B-2020Antwerpen Belgium; e Belgian Nuclear Research Centre (SCK CEN) Institute for Nuclear Energy Technology B-2400Mol Belgium; f Rossendorf Beamline, ESRF – The European Synchrotron, CS40220, 38043Grenoble Cedex 9, France; g Institute of Resource Ecology Helmholtz Zentrum Dresden-Rossendorf (HZDR) PO Box 510119 01314Dresden Germany; University College London, United Kingdom

**Keywords:** X-ray absorption spectroscopy, HERFD-XANES, electronic structure, *FDMNES*

## Abstract

Here, the uranium valence electronic structures in the perovskite-based ternary uranate systems NaUO_3_, KUO_3_ and RbUO_3_ are reported on the basis of high-energy resolved fluorescence-detected X-ray absorption spectroscopy experiments at the U *L*_3_ edge and relativistic quantum chemistry calculations based on density functional theory. Advanced theoretical simulations allowed us to identify the origin of spectral features and to assess the impact of structural distortion within the oxygen octahedra. The octahedral crystal-field strength extracted from both experiments and calculations is reported for all three compounds.

## Abstract 2

High-energy resolved fluorescence-detected X-ray absorption spectroscopy at the uranium *L*_3_ edge and relativistic quantum chemistry calculations are combined to study in detail the uranium valence electronic structure in NaUO_3_, KUO_3_ and RbUO_3_. The impact of the structural distortion of the oxygen octahedra around uranium on the contribution of uranium and oxygen states in observed spectral features is discussed.

## 1. Introduction

Uranium compounds have been triggering scientific interest for many decades, not only for their nuclear energy applications but also for more fundamental aspects. Indeed, uranium shows very versatile physicochemical properties due to the wide range of its possible oxidation states, and due to the rather complex behavior of its 5*f* electrons, the origins of which are not yet clearly understood. Uranium 5*f* electrons show an apparent duality in localization, being often found in radially dispersed and hybridized bands in the vicinity of the Fermi level, whereas sometimes they remain localized (Guziewicz *et al.*, 2004 ▸; Teterin *et al.*, 1981 ▸; Teterin & Teterin, 2004 ▸). Consequently, they can simultaneously participate in the conduction band and remain localized, producing a mixed covalent/ionic character of the uranium bonding (Kaltsoyannis, 2013 ▸). When working with a significant amount of uranium, dedicated laboratories or specific safety measures are usually required to safely handle the natural radioactivity of uranium, but despite that a large amount of crystallographic, physical, chemical and thermodynamic data is available today (Grenthe *et al.*, 2006 ▸); however, the uranium electronic structure is not yet completely understood. The experimental difficulties are also reflected in the complexity of accurate theoretical calculations because of comparable magnitudes of the crystal field, spin–orbit coupling and the electron–electron repulsion interactions affecting simultaneously the electronic structure. Fortunately, one can significantly reduce such a complexity by exploring pure pentavalent U(V) compounds, where no electron–electron repulsion interactions are expected within the 5*f* shells.

The pentavalent U(V) state has essentially been identified in oxidation products of UO_2_, such as U_4_O_9_, U_3_O_7_ and U_3_O_8_. However, with the exception of the pure pentavalent U_2_O_5_ phase, which has been reported to exist only under very specific conditions (Hoekstra *et al.*, 1970 ▸; Gouder *et al.*, 2018 ▸), pentavalent uranium always occurs as a mixture with tetra- or hexa-valent uranium in those compounds (Kvashnina *et al.*, 2014 ▸; Leinders *et al.*, 2017 ▸, 2021 ▸).

Pure pentavalent uranium compounds are also commonly observed in ternary systems of uranium and oxygen with one additional cation, *e.g.* from the alkali metal group, or certain alkaline earth and transition metals, as well as some of the rare earth elements (Selbin & Ortego, 1969 ▸). Often stable, these ternary compounds may sometimes suffer from slight oxygen non-stoichiometry, leading to a mixed-valence character (Grenthe *et al.*, 2006 ▸).

Within the alkali metal uranates *M*UO_3_ series (with *M* being Li, Na, K, Rb and Cs), a pure U(V) valence state has been confirmed by experimental and theoretical studies for Li, Na, K and Rb, while the CsUO_3_ compound was reported as non-existing (Kovba & Golubenko, 1960 ▸; Aravamudan *et al.*, 1978 ▸; Cordfunke & Ouweltjes, 1981 ▸; Chippindale *et al.*, 1989 ▸; Ball, 1992 ▸; Soldatov *et al.*, 2007 ▸; Misra *et al.*, 2008 ▸; Liu *et al.*, 2009 ▸; Azam & Reshak, 2014 ▸; Butorin *et al.*, 2016 ▸; Leinders *et al.*, 2017 ▸; Lopez *et al.*, 2017 ▸; Sanyal *et al.*, 2017 ▸; Dorbane *et al.*, 2019 ▸; Leinders *et al.*, 2020 ▸; Bes *et al.*, 2022 ▸).

**Figure 1:**
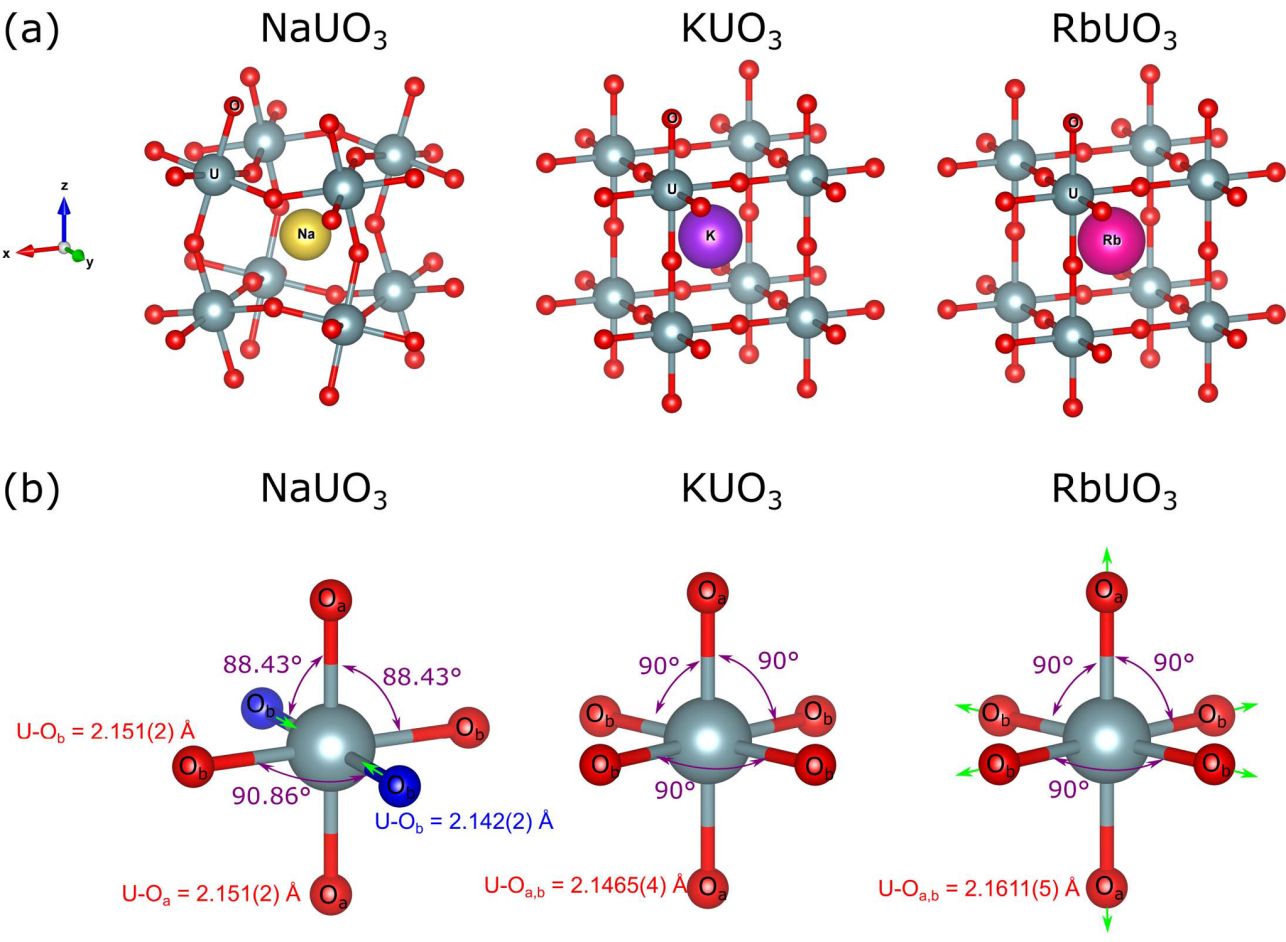
(*a*) Representation of the cubic arrangement of the eight nearest UO_6_ bi-pyramids around the alkali metals in NaUO_3_, KUO_3_ and RbUO_3_. The projection view is along the normal of the (723) plane. (*b*) Representation of the oxygen bi-pyramid around uranium for NaUO_3_, KUO_3_ and RbUO_3_. For an easy comparison between all three uranates, all the apices are oriented along *z*, *i.e.* without the expected tilt shown in (*a*), but the rotation of the base around the *z* axis is shown. The distortion in terms of U–O distances (contraction and elongation) is marked with light green arrows, relative to KUO_3_. Nonequivalent distances between uranium and each of the oxygen atoms forming the apices O_a_ and the base O_b_ of the bi-pyramid are shown, using the same color as the oxygen atoms they refer to (*e.g.* blue and red O_b_ in the case of NaUO_3_). Main angles between atoms are also indicated. This figure was partially created using the *VESTA* 3 software (Momma & Izumi, 2011 ▸).

The series is related to perovskites, as suggested by their chemical formulae *M*UO_3_, and shows an interesting gradation in structural property and stability. The stability of perovskites is often discussed in terms of a Goldschmidt derived tolerance factor *t*, based only on the chemical formula, here *M*UO_3_, and the ionic radii, *r*_i_, of each ion (*M*, U, O). This tolerance factor is given by
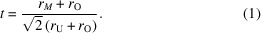
The closer *t* is to unity, the more stable the regular perovskite structure is, while distorted variants are more likely when *t* is deviating from unity. Using Shannon’s crystal radii for six-coordinated uranium and oxygen, and 12-coordinated alkali, tolerance factors of 0.87, 0.91, 1.00, 1.02 and 1.07 are reported for *M*UO_3_ perovskites with *M* being Li, Na, K, Rb and Cs, respectively (Ball, 1992 ▸; Bartel *et al.*, 2019 ▸). CsUO_3_ has a tolerance factor that is significantly greater than unity. As discussed by Ball (1992 ▸), one would thus expect alternative structures such as that of hexagonal-close-packed BaNiO_3_ to be adopted, but no stable CsUO_3_ has been reported yet. Having a tolerance factor very close to unity, KUO_3_ and RbUO_3_ both crystallize in a prototypical undistorted perovskite-type structure in space group 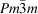, but differ in the value of the lattice constant, *i.e.* 4.2930 (6) Å for KUO_3_ and 4.3222 (9) Å for RbUO_3_. The U–O distances are thus slightly longer in RbUO_3_ than in KUO_3_ with 2.1611 (5) Å and 2.1465 (4) Å, respectively (Van den Berghe *et al.*, 2004 ▸). However, NaUO_3_ and LiUO_3_ have values of *t* that are significantly less than unity, leading to distorted structures. LiUO_3_ adopts the lithium niobate structure, which can be regarded as a heavily distorted perovskite structure, while NaUO_3_ crystallizes in an orthorhombically distorted perovskite structure, described in space group *Pbnm*, with lattice constants *a* = 5.7739 (2) Å, *b* = 5.9051 (2) Å, and *c* = 8.2784 (2) Å. The bi-pyramid geometry of the oxygen octahedra observed in KUO_3_ and RbUO_3_ is slightly distorted in NaUO_3_, forming an oblique bi-pyramid with a parallelogram base (Van den Berghe *et al.*, 2004 ▸). For the sake of clarity, Fig. 1 ▸ compares the structural changes (bond length and angles) observed in the oxygen octahedra between NaUO_3_, KUO_3_ and RbUO_3_. In NaUO_3_, the distances between uranium and two opposite oxygen atoms of the base O_b_ are 2.142 (2) Å and 2.151 (2) Å, the latter being equal to the distance between uranium and each of the oxygen atoms forming the apices of the bi-pyramid O_a_. The square base of the regular bi-pyramid is not maintained, but distorted into a parallelogram with angles of 89.14 (3)° and 90.86 (3)°, instead of 90°. As a result, angles between the four atoms of the base and the apices of the bi-pyramid, O_b_–U–O_a_, are no longer 90° but 91.44 (8)°, 91.57 (8)°, 88.43 (8)° and 88.56 (8)°. However, the angles between two opposite oxygen atoms relative to uranium are still equal to 180°.

The bond length and angle distortions of the oxygen octahedra are likely to affect the strength of the crystal field, producing noticeable changes in the uranium electronic structure. Indeed, when the ligand–metal bond length increases, the crystal-field splitting energy generally decreases owing to the overlap between the metal *d*-orbitals and the ligand orbitals becoming weaker as the distance increases. Similarly, structural distortions likely weaken the metal–ligand orbital overlapping and thus the crystal-field splitting. Therefore, by studying the uranium electronic structure behavior along the alkali *M*UO_3_ series, we are aiming at a better understanding of the electronic properties of uranium. In this paper, we report the results of our study on the uranium valence electronic structure in the prototypical perovskite systems KUO_3_ and RbUO_3_, and in the distorted perovskite NaUO_3_, by means of uranium *L*_3_-edge high-energy resolution/resolved fluorescence-detected X-ray absorption spectroscopy (HERFD-XAS) and state-of-the-art relativistic quantum chemistry calculations based on density functional theory (DFT). The results are found to complement the insights obtained previously on KUO_3_ (Bes *et al.*, 2022 ▸) using multi-edge HERFD-XAS (uranium *L*_1_, *L*_3_ and *M*_4_ edges) combined with DFT calculations.

## 2. Materials and methods

### 2.1. Sample preparation

Polycrystalline powders of NaUO_3_, KUO_3_ and RbUO_3_ were prepared following the same methodology for all compounds. Firstly, a batch of U_3_O_8_ powder was prepared by treating depleted nuclear-grade (according to ASTM C 753-04) UO_2+*x*_ powder at 500°C for 4 h in a muffle furnace under normal atmospheric conditions (N_2_/21 vol.% O_2_). Stoichiometric amounts of U_3_O_8_ were then intimately mixed with the carbonate powder of each respective alkali metal, *i.e.* NaCO_3_, KCO_3_ and RbCO_3_ (≥99.0% purity according to ACS Reagent), in a small excess (2 wt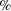), using a zirconia mortar and pestle. Each powder mixture was transferred into Al_2_O_3_ crucibles and an annealing was performed in a tubular furnace at 800°C for 10 h. A reducing atmosphere (−400 kJ mol^−1^ at 800°C) was applied by flushing the furnace with a gas mixture of Ar/0.5 vol.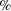 O_2_ (519 ml min^−1^) and Ar/5 vol.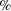 H_2_ (481 ml min^−1^). The used gases were of high purity (99.9992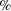) and flow rates were accurately controlled using Bronkhorst EL-FLOW mass-flow controllers.

X-ray powder diffraction was performed on each sample to verify phase purity. A PANalytical X’Pert Pro equiped with a Cu LFF X-ray tube and operating in Bragg–Brentano geometry was used. The beam path consisted of a fixed divergence slit (0.5°), a 10 mm beam mask, 0.02 rad Soller slit assemblies and a Ni filter. Diffraction patterns were measured over the angular range 19–144° 2θ using a position-sensitive detector (PANalytical X’Celerator) with a window of 2.2° 2θ. Data assessment, including Rietveld refinement, was performed using the *HighScorePlus* (v4) software. The diffraction patterns obtained from each sample were in accordance with the corresponding calculated patterns from the crystallographic information reported in the literature (Van den Berghe *et al.*, 2004 ▸). Small amounts of a UO_2_ impurity phase could be detected in the powder samples of NaUO_3_ (3 ± 1 wt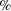) and RbUO_3_ (5 ± 1 wt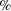). No impurity phase could be detected in the KUO_3_ powder sample. The diffraction patterns and resulting Rietveld fits of each sample are available in the supporting information. In the supporting information, a qualitative study of the UO_2_ impurity effect on the high-energy resolution fluorescence-detected X-ray absorption near-edge structure (HERFD-XANES) spectral features is available. It demonstrates that the presence of this impurity does not affect the conclusions drawn hereafter.

### 2.2. High-energy resolution fluorescence-detected X-ray absorption spectroscopy

Uranium *L*_3_-edge XAS measurements were performed at the Rossendorf Beamline (BM20) (Matz *et al.*, 1999 ▸; Scheinost *et al.*, 2021 ▸) of the European Synchrotron Radiation Facility (ESRF) operating at an electron-beam energy of 6 GeV in Grenoble, France. The incident energy was scanned using a Si(311) monochromator. HERFD-XAS spectra were collected at room temperature using an X-ray emission spectrometer equipped with five Si(220) crystal analyzers with 1 m bending radius, and a silicon drift X-ray detector in a vertical Rowland geometry (Kvashnina & Scheinost, 2016 ▸). The spectrometer was tuned to the maximum of the U *L*_3_O_4,5_ (*L*β_5_, 2*p*_3/2_–5*d*_5/2_ transition at 17.063 keV) X-ray emission line using the 10 10 0 reﬂection at a Bragg angle of 71° (Leinders *et al.*, 2025 ▸). The detected intensity was normalized to the incident ﬂux. The estimated beam size was 50 µm (vertically) and 2 mm (horizontally). A total experimental energy broadening (incident energy convoluted with emitted energy and core-hole lifetime broadening) of 2.2 eV was achieved, well below the core-hole lifetime broadening of the U *L*_3_ edge (∼8.2 eV). This value is greatly improved compared with the 3.6 eV achieved in our previous study using a Si(111) monochromator and collecting the U *L*_3_*M*_5_ emission line (*L*α_1_, 2*p*_3/2_–3*d*_5/2_ transition) (Bes *et al.*, 2022 ▸). During the experiment reported here, spectra were also collected using the U *L*_3_*M*_5_ emission line for comparison, using the 880 reﬂection at a Bragg angle of 72°. Energy calibration was achieved through the *K*-edge excitation energy (17.038 keV) of a metallic yttrium foil placed in the beam path. The *M*UO_3_ powder was directly inserted and sealed with Kapton foil inside a small recess of a polypropylene sample holder. The total measurement time was ∼3 h per sample. The integration time was 5 s per point, and 30-min long scans were repeated six times and then merged.

### 2.3. Electronic structure calculations

Relativistic quantum chemistry calculations were performed using the *Finite Difference Method for Near-Edge Structure* (*FDMNES*) code (Bunău & Joly, 2009 ▸). Atomic clusters of 9 Å were used in self-consistent-ﬁeld calculations using the Dirac–Slater approach. The Poisson equation was solved to obtain the Coulomb potential from the superposed self-consistent atomic densities in the considered cluster. The energy-dependent exchange–correlation potential was evaluated using the local density approximation, and constructed using both the real Hedin–Lundqvist and Von Barth formulations. These calculations were based on static atom supercells of hundreds of atoms, and thermally induced disorder was not considered. Because of the presence of heavy nuclei (U), spin–orbit effects were taken into account, but no spin-polarization effect was noticed from the comparison of spin-polarized spectra. Finally, calculations were performed with and without the quadrupolar transition probability in order to account for the 5*f* contribution on the ﬁnal spectra at the U *L*_3_ edge.

## 3. Results and discussion

**Figure 2:**
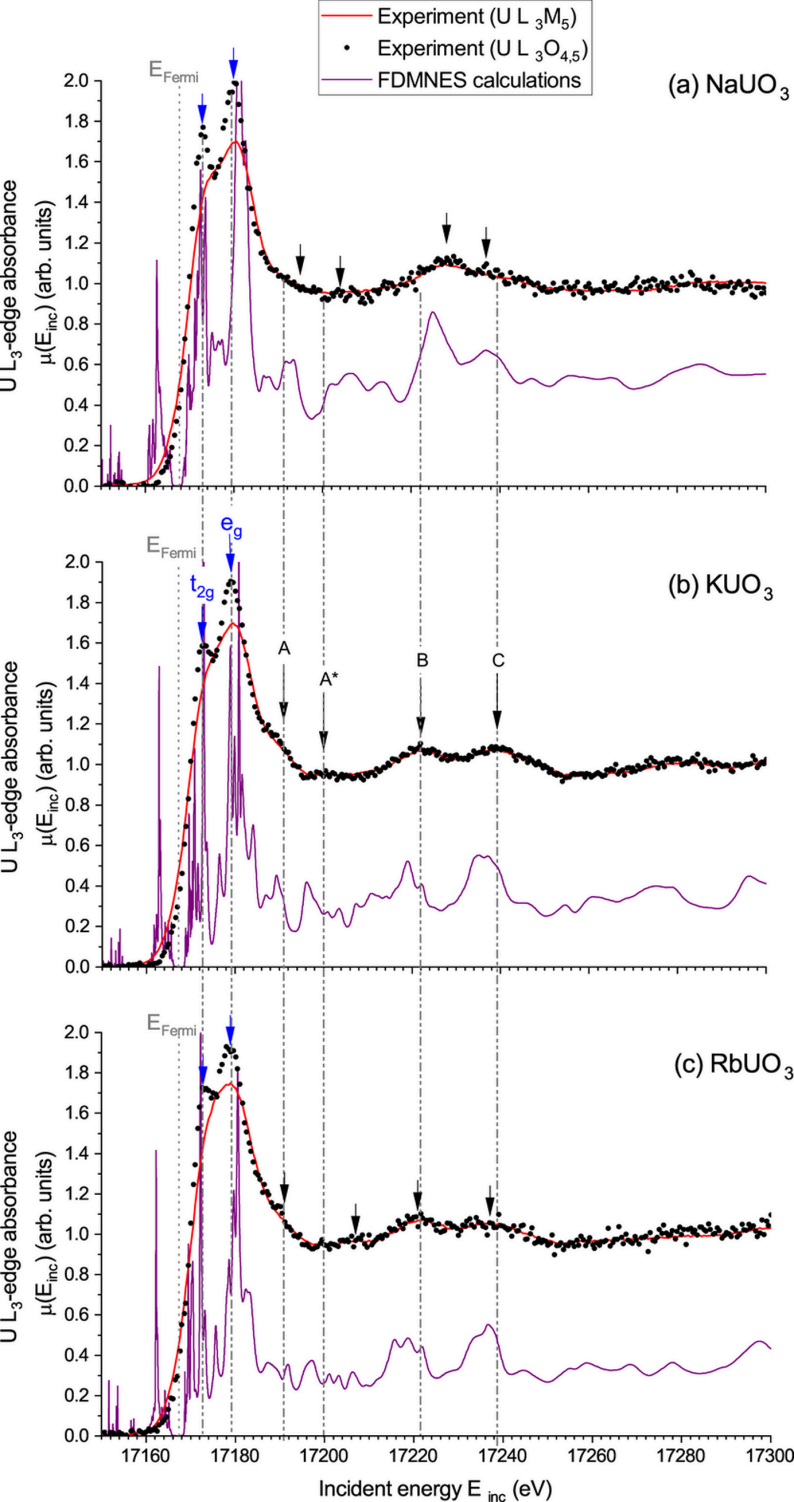
Uranium *L*_3_-edge HERFD-XAS and the corresponding simulated spectrum using *FDMNES* for NaUO_3_, KUO_3_ and RbUO_3_. The calculated spectra account for both dipolar and quadrupolar transitions (see the supporting information for comparison of simulated spectra with and without the quadrupolar transition). The Fermi energy level is indicated with a vertical dotted line. Vertical dashed–dotted lines indicate the positions of features A, A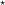, B and C, as experimentally observed in KUO_3_, to guide the eye. Blue arrows highlight the crystal-field splitted U-*d* orbitals (t_2g_ and e_g_), while black arrows indicate the positions of the post-edge observed features.

Fig. 2 ▸ shows the obtained HERFD-XAS spectra of NaUO_3_, KUO_3_ and RbUO_3_ using the U *L*_3_O_4,5_ emission line, together with the spectra collected using the U *L*_3_*M*_5_ emission line and the simulated spectra using the *FDMNES* code. Superimposed experimental spectra, as well as their comparison with their corresponding simulated but convoluted spectra, are available in the supporting information.

Thanks to the tremendous gain in energy resolution when using the U *L*_3_O_4,5_ emission line compared with the spectra collected using the U *L*_3_*M*_5_ emission line, the crystal-field splitting of the uranium 6*d* orbitals is well resolved in all three uranates. For all three compounds, the maximum of the white line occurs at 17172.9 ± 0.5 eV, while the maximum of the first derivative, which corresponds to the uranium *L*_3_-edge energy position *E*_0_, is found at 17169.9 ± 0.5 eV. Therefore, no energy shift in the edge position is observed between all three compounds.

The occurrence of a quadrupolar transition of 2*p* → (*n*)*f* has been previously reported on many uranium compounds. Vitova *et al.* (2010 ▸) first observed such a transition as a shoulder in the rising edge on UO_2_, [UO_2_py_5_][KI_2_py_2_] and UO_2_(NO_3_)_2_(H_2_O)_6_. Later on, Kvashnina *et al.* (2014 ▸) reported a similar observation on U_4_O_9_ and U_3_O_8_, while the same observation was also made on [Ni(H_2_O)_4_]_3_[U(OH,H_2_O)(UO_2_)_8_O_12_(OH)_3_] (Bès *et al.*, 2016 ▸). Nevertheless, no quadrupolar transition is observed in our experimental spectra. As previously discussed for KUO_3_ (Bes *et al.*, 2022 ▸), this is essentially a consequence of both the partial overlap between U-5*f* and U-6*d* electrons, being also separated by about 2 eV in NaUO_3_ and RbUO_3_, and the weak strength of the quadrupolar transition relative to the dipolar one, as confirmed by our calculations (see the supporting information). The weak quadrupolar feature thus remains hidden within the white line for all three uranates.

The splitting of the U-6*d* orbitals follows the expected trends when comparing KUO_3_ and RbUO_3_: the crystal-field strength is weakened when increasing the ligand–metal distance. However, if the structural distortions in NaUO_3_ are expected to break the symmetry of the O octahedra, this will reduce the strength of the crystal-field splitting. Nevertheless, the symmetry break is probably not enough in NaUO_3_ because one may observe a slight increase of the U-6*d* splitting when comparing KUO_3_ and NaUO_3_ spectra. This increase is the probable consequence of the shortening of some of the ligand–metal distances, having more effect on the crystal-field strength compared with the bonding angle distortions.

In addition to the split white line, several features are clearly visible in the post-edge region. The same features A, A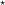, B and C, as already reported earlier for KUO_3_ (Bes *et al.*, 2022 ▸), are also observed in NaUO_3_ and RbUO_3_, but they appear at slightly different energy positions. All these observed spectral features are reproduced by the calculations in terms of number and relative intensity, but with a few electronvolt discrepancies on their positions. Therefore, a very good agreement between experiments and calculation is demonstrated. A comparison between the experimentally observed and calculated energy positions of the spectral features is reported in Table 1 ▸.

The spectra of KUO_3_ and RbUO_3_ are very close to each other, as expected given their structural similarities. The longer U–O distance, however, seemingly affects the position of the A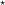 feature, shifting it to higher energies by 8 eV relative to A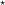 in KUO_3_. A closer look at the calculated spectra gives another explanation for such a surprisingly large energy shift. Indeed, a weak feature situated nearby the expected energy is observed, which is likely to be the missing A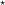 feature. A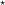 is probably not observed in the experimental spectrum of RbUO_3_ because of the occurrence of a new feature, A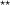, situated about 10 eV above A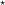 and because of statistical uncertainties. In addition to their weak intensities, the energy difference between the calculated positions of A and A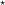 is reduced to 5.3 eV compared with the 7.2 eV observed in KUO_3_, making them more difficult to distinguish. Structural distortion in NaUO_3_ has a stronger effect on the spectral features. A and A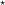 are shifted to higher energies but remain separated by about 9 eV. B is also shifted to higher energies while C is moved to lower energies, reducing their energy difference from ∼17 to 9 eV. The energy differences of the positions of the spectral features for NaUO_3_ and RbUO_3_ relative to KUO_3_ are reported in Table 2 ▸. In this table, despite the large uncertainties associated with some reported values, the effect of the structural distortion in NaUO_3_ is clearly visible for the A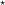 and B features, where values of Δ_exp_ and Δ_cal_ are significantly different from 0.

**Figure 3:**
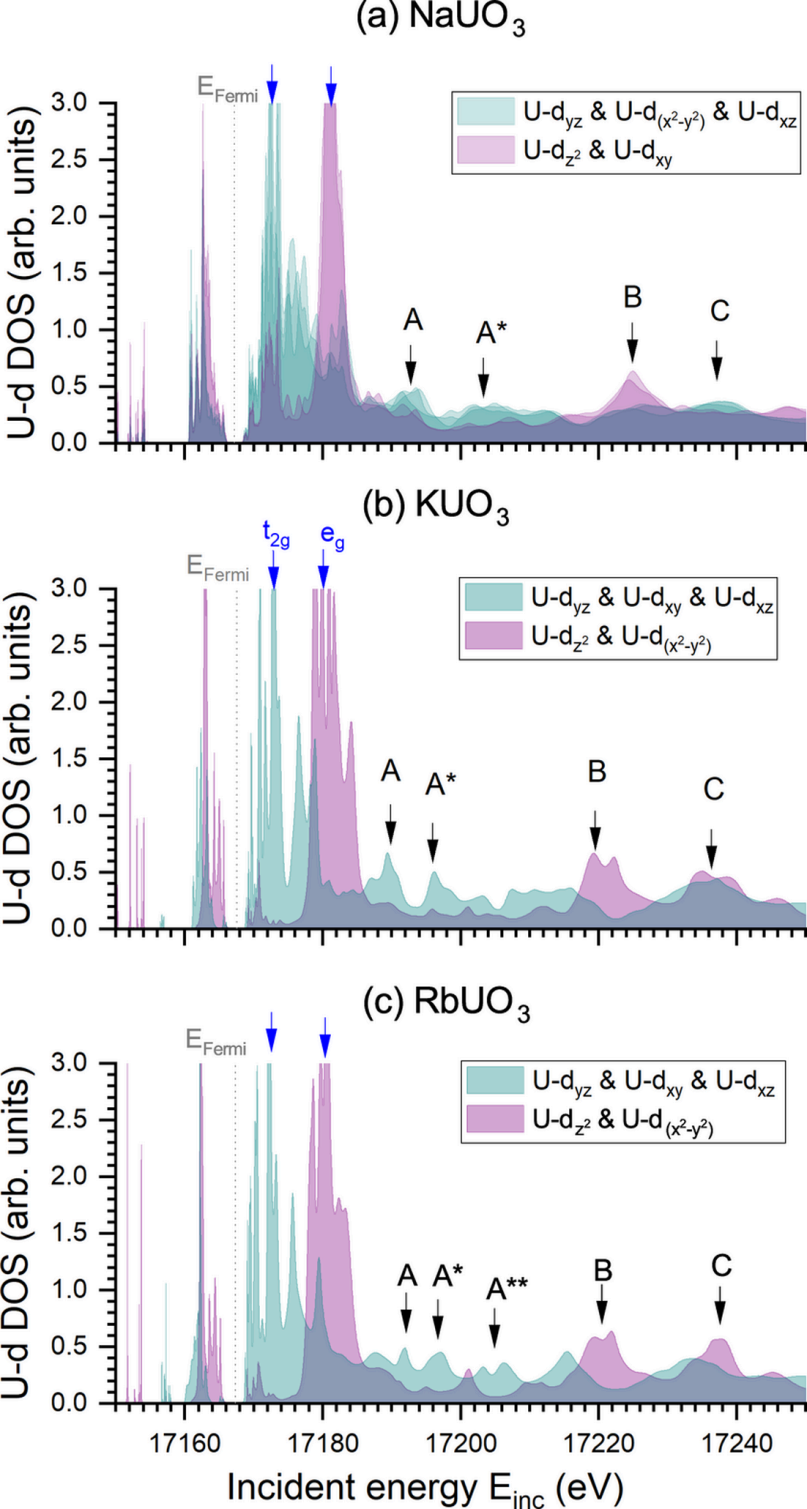
Calculated U-*d* partial density of states (DOS), here expressed as cubic harmonics, for (*a*) NaUO_3_, (*b*) KUO_3_ and (*c*) RbUO_3_. The Fermi energy level is indicated with a vertical dotted line. Blue arrows highlight the crystal-field splitted U-*d* orbitals (t_2g_ and e_g_). The positions of the post-edge features A, A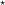, A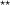, B and C from the simulated spectra are indicated by black arrows.

**Figure 4:**
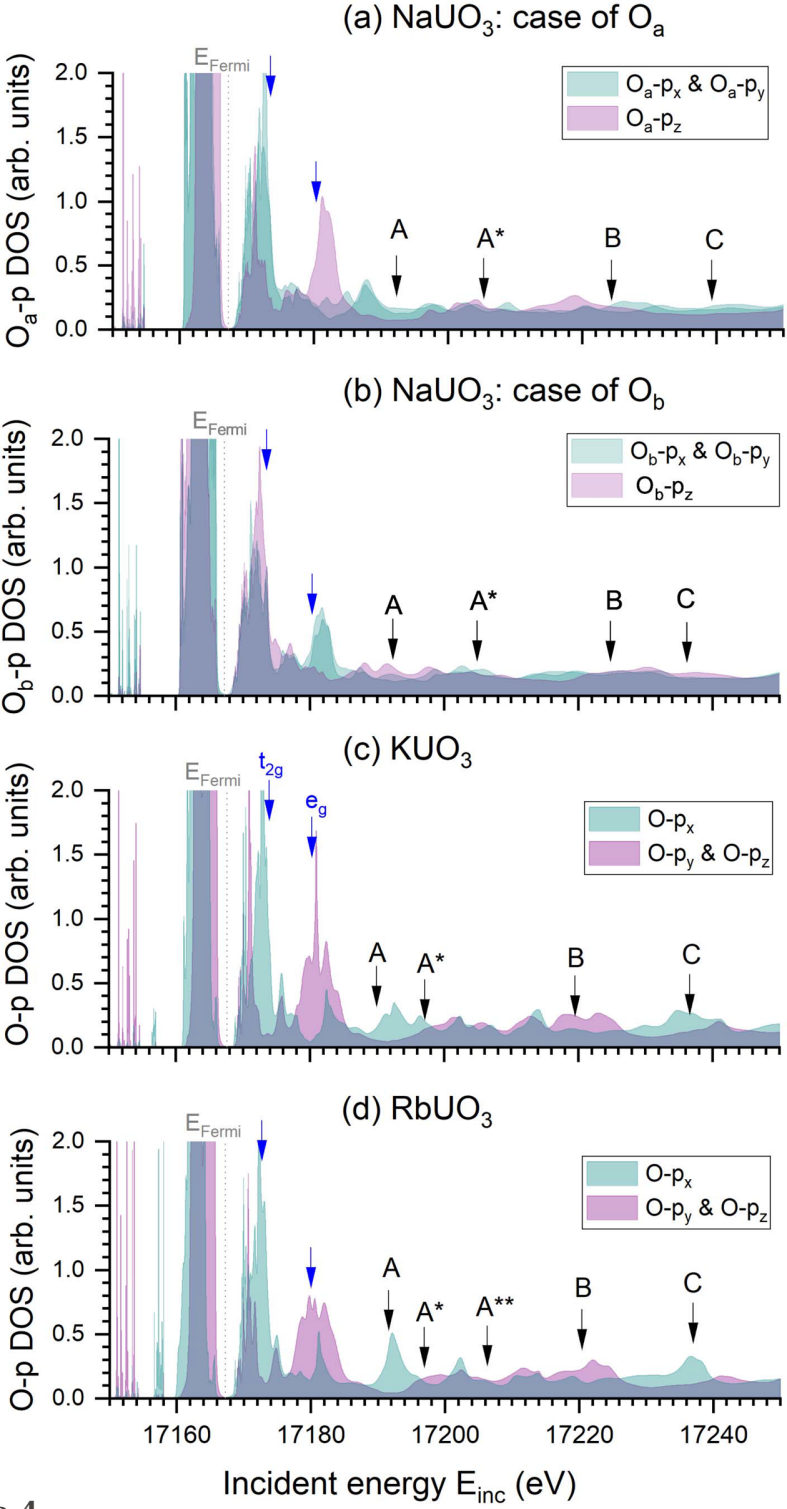
Calculated O-*p* partial density of states, here expressed as cubic harmonics, for (*a*) the apices oxygen O_a_ from NaUO_3_, (*b*) the base oxygen O_b_ from NaUO_3_, (*c*) KUO_3_ and (*d*) RbUO_3_. The Fermi energy level is indicated with a vertical dotted line. Blue and black arrows highlight the calculated positions of the spectral features from Fig. 3 ▸.

Figs. 3 ▸ and 4 ▸ show the calculated U-*d* and O-*p* density of states, respectively. We refer to the supporting information for the other U-related density of states, as well as the counter-ion density of states. To gain more insight into the nature of the spectral features, these partial densities of states are represented as cubic harmonics. The U-*d* and O-*p* orbitals involved in each of the features are reported in Table 3 ▸, where individual orbitals are mentioned only when a significant contribution is observed in Fig. 3 ▸ or in Fig. 4 ▸. When considering the crystal field in an octahedral geometry, the U-*d* orbitals are divided into two degenerated groups: t_2g_, composed of U-*d*_*xy*_, U-*d*_*yz*_ and U-*d*_*yz*_ orbitals; and e_g_, composed of U-*d*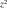 and U-*d*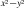 orbitals. The former is pulled to lower energy, while the latter is pushed to higher energy. These two groups are clearly visible for KUO_3_ and RbUO_3_ for which a perfect overlap of the orbitals expected in each group is observed in the calculations. For NaUO_3_, the perfect octahedral symmetry is broken as a consequence of the structural distortion, releasing the degeneracy of the orbitals initially involved in t_2g_ and e_g_. However, such a release is not very strong, and two groups of almost overlapping orbitals remain visible despite the occurrence of a larger density of states observed in between t_2g_ and e_g_. Another surprising finding is the group exchange of the U-*d*_*xy*_ and U-*d*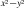 orbitals. One plausible explanation for this exchange is found by considering that the octahedra are approximately rotated by 45° around the *z* axis (*i.e.* the O_a_–O_a_ axis) when passing from KUO_3_ to NaUO_3_. The main difference between the U-*d*_*xy*_ and U-*d*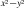 orbitals originates from the directions where the lobes are pointing to: along the referential *x* and *y* axes or in between. Therefore, a 45° rotation is switching from one to the other.

The crystal-field strength, 10Dq, can be deduced from the energy separation between t_2g_ and e_g_ (see Table 1 ▸). Corresponding experimental values of 6.8 ± 1 eV, 6.3 ± 1 eV and 6.2 ± 1 eV, as well as calculated values of 6.8 ± 0.4 eV, 6.9 ± 0.4 eV and 7.3 ± 0.4 eV, were estimated for NaUO_3_, KUO_3_ and RbUO_3_, respectively. These values do not clearly differ from each other within the uncertainty range. Thus, no significant effect of the longer U–O distance or the slight structural distortion of the octahedra are noticeable. This is in line with the experimental and theoretical crystal-field strengths of 6 ± 2 eV and 8.2 ± 0.4 eV, respectively, reported in FeUO_4_, which exhibits strong structural distortion of the oxygen octahedra (Yomogida *et al.*, 2022 ▸). However, on the sole basis of our calculations, the effects of the spin–orbit coupling, the *p*–*d* multiplet interaction or the 2*p* core hole in the observed splitting cannot be ruled out and may somehow compensate the changes in the crystal field. Further theoretical studies are therefore required to unravel the potentially intricate roles of these effects and the crystal field in these uranates.

If no significant difference is surprisingly observed in the U-6*d* splitting strength of all three uranates, the electronic structure still differs in the post-edge. According to the calculations, feature A originates mainly from U-*d* states U-*d*_*yz*_, U-*d*_*xy*_ and U-*d*_*xz*_ for KUO_3_ and RbUO_3_; and from U-*d* states U-*d*_*yz*_, U-*d*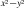 and U-*d*_*xz*_ for NaUO_3_. No significant contribution from O-*p* states is noticed for KUO_3_, but the O_b_-*p*_*z*_ state slightly contributes for NaUO_3_ and the O-*p*_*x*_ state strongly contributes for RbUO_3_. Feature A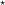 also originates mainly from U-*d* states, with no significant contribution from O-*p* states in KUO_3_ and RbUO_3_. In RbUO_3_, A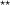 is similar to A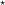. In NaUO_3_, slight contributions of the O_b_-*p*_*x*_, O_b_-*p*_*y*_ and O_a_-*p* states are observed in A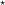. Feature B is essentially related to U-*d*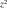 and U-*d*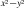 (U-*d*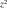 and U-*d*_*xy*_ for NaUO_3_), with also a significant contribution of O-*p*_*x*_ in KUO_3_ and RbUO_3_. In the case of NaUO_3_, the O-*p* contribution is less clear, with no significant peak observed in the O-*p* density of states, except for O_a_-*p*_*x*_. Feature C appears to result from a mixture of all U-*d* electrons in KUO_3_, but this is not the case for RbUO_3_ and NaUO_3_. In the former compound, U-*d*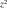 and U-*d*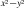 start to dominate, while in the latter compound, it is U-*d*_*yz*_, U-*d*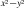 and U-*d*_*xz*_ that dominate. Moreover, contributions of O-*p*_*y*_ and O-*p*_*z*_ are observed for KUO_3_ and RbUO_3_. The contribution of O-*p* states in NaUO_3_ is less clear, but a probable inversion is observed between O_b_ and O_a_ in terms of which orbital contributes. The U-*s*, U-*p* and U-*f* orbitals were found to not have a significant contribution to the observed features (see the supporting information). Similarly, the potential binding interactions of the counter ion were ruled out based on their calculated density of states (available as well in the supporting information), with no significant energy overlap between the observed spectral features and the calculated density of states. This result was also confirmed through comparison of the calculated spectra of NaUO_3_ and RbUO_3_ with and without the counter ion replaced by K without changing the structure of the uranates (also available in the supporting information).

## 4. Conclusions

Here, we reported HERFD-XAS data obtained at the uranium *L*_3_ edge of NaUO_3_, KUO_3_ and RbUO_3_. By collecting the U *L*_3_O_4,5_ emission line, an overall resolution of 2.2 eV was achieved, allowing the separation of the split U-6*d* orbitals to become visible. The experimental data were compared with theoretical calculations to identify the nature of the U-*d* and O-*p* orbitals contributing to the observed spectral features. These calculations are in very good agreement with the experiments. More insight into the nature of the spectral post-edge features was obtained by using U and O partial density of states projected into cubic harmonics. The strength of the crystal-field splitting of the U-6*d* states was evaluated experimentally to 6.8 ± 1 eV, 6.3 ± 1 eV and 6.2 ± 1 eV, and theoretically to 6.8 ± 0.4 eV, 6.9 ± 0.4 eV and 7.3 ± 0.4 eV, for NaUO_3_, KUO_3_ and RbUO_3_, respectively. No significant difference was observed here despite the structural differences existing between all three uranates. One possible hypothesis is that the crystal-field strength may be compensated due to opposite effects on orbitals by, for example, spin–orbit coupling, *p*–*d* multiplet interaction or the 2*p* core hole. If preliminary studies tend to demonstrate that the core hole slightly enhances the crystal field, the reason behind our observations remains to be clarified by further studies, supported by more advanced theories. The four post-edge features already observed in KUO_3_ are also present in NaUO_3_ and RbUO_3_, but their character in terms of U-*d* and O-*p* cubic orbital mixtures is affected by the U–O distance and the structural distortion of the oxygen octahedra around uranium atoms.

## Supplementary Material

Supporting information. DOI: 10.1107/S1600577525005156/ing5005sup1.pdf

## Figures and Tables

**Table 1:** Energy positions of experimentally observed and calculated uranium *L*_3_-edge HERFD-XAS spectral features for NaUO_3_, KUO_3_ and RbUO_3_ Differences between the experimental and calculated values, Δ_exp−cal_, are also given for convenience.

Uranate	Feature	Experimental	Calculated	Δ_exp−cal_
NaUO_3_	t_2g_	17172.9 ± 0.5 eV	17173.5 ± 0.2 eV	−0.6 eV
	e_g_	17179.7 ± 0.5 eV	17180.3 ± 0.2 eV	−0.6 eV
	A	17195 ± 1 eV	17192.3 ± 0.2 eV	2.7 eV
	A 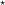	17204 ± 1 eV	17204.9 ± 0.2 eV	−0.9 eV
	B	17228.0 ± 0.5 eV	17224.8 ± 0.2 eV	3.2 eV
	C	17236.9 ± 0.5 eV	17237.3 ± 0.2 eV	−0.4 eV
KUO_3_	t_2g_	17172.9 ± 0.5 eV	17173.3 ± 0.2 eV	−0.4 eV
	e_g_	17179.2 ± 0.5 eV	17180.2 ± 0.2 eV	−1.0 eV
	A	17191 ± 1 eV	17189.9 ± 0.2 eV	1.1 eV
	A 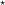	17200 ± 1 eV	17197.1 ± 0.2 eV	2.9 eV
	B	17222.0 ± 0.5 eV	17219.5 ± 0.2 eV	2.5 eV
	C	17239.2 ± 0.5 eV	17236.6 ± 0.2 eV	2.6 eV
RbUO_3_	t_2g_	17172.9 ± 0.5 eV	17172.7 ± 0.2 eV	0.2 eV
	e_g_	17179.1 ± 0.5 eV	17180.0 ± 0.2 eV	−0.9 eV
	A	17191 ± 1 eV	17191.6 ± 0.2 eV	−0.6 eV
	A 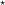	–	17196.9 ± 0.2 eV	–
	A 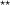	17207 ± 1 eV	17206.3 ± 0.2 eV	0.7 eV
	B	17220.9 ± 0.5 eV	17220.4 ± 0.2 eV	0.4 eV
	C	17237.1 ± 0.5 eV	17237.0 ± 0.2 eV	0.1 eV

**Table 2:** Energy difference of experimentally observed, Δ_exp_, and calculated, Δ_cal_, spectral features for NaUO_3_ and RbUO_3_ relative to KUO_3_ Uncertainties are ±1 and ±0.4 eV for experiments and calculations, respectively.

Uranate	Feature	Δ_exp_	Δ_cal_
NaUO_3_	t_2g_	0 eV	0.2 eV
	e_g_	0.5 eV	0.1 eV
	A	1 eV	2.4 eV
	A 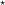	4 eV	7.8 eV
	B	6 eV	5.3 eV
	C	2.3 eV	0.7 eV
RbUO_3_	t_2g_	0 eV	−0.6 eV
	e_g_	−0.1 eV	−0.2 eV
	A	1 eV	1.7 eV
	A 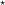	–	−0.2 eV
	B	−1.1 eV	0.9 eV
	C	−2.1 eV	0.4 eV

**Table 3:** The U-*d* and O-*p* cubic harmonics contributing to the spectral features

Uranate	Feature	Main U-*d* cubic harmonics	Main O-*p* cubic harmonics
KUO_3_	t_2g_	U-*d*_*xy*_, U-*d*_*xz*_, U-*d*_*yz*_	O-*p*_*x*_
	e_g_	U-*d* 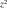 , U-*d* 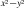	O-*p*_*y*_, O-*p*_*z*_
	A	U-*d*_*xy*_, U-*d*_*xz*_, U-*d*_*yz*_	O-*p*_*x*_
	A 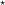	U-*d*_*xy*_, U-*d*_*xz*_, U-*d*_*yz*_	–
	B	U-*d* 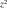 , U-*d* 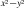	O-*p*_*y*_, O-*p*_*z*_
	C	All	O-*p*_*x*_
NaUO_3_	t_2g_	U-*d* 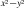 , U-*d*_*xz*_, U-*d*_*yz*_	O_a_-*p*_*x*_, O_a_-*p*_*y*_, O_b_-*p*_*z*_
	e_g_	U-*d* 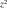 , U-*d*_*xy*_	O_b_-*p*_*x*_, O_b_-*p*_*y*_, O_a_-*p*_*z*_
	A	U-*d* 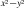 , U-*d*_*xz*_, U-*d*_*yz*_	O_b_-*p*_*z*_
	A 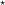	U-*d* 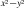 , U-*d*_*xz*_, U-*d*_*yz*_	–
	B	U-*d* 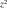 , U-*d*_*xy*_	–
	C	U-*d* 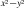 , U-*d*_*xz*_, U-*d*_*yz*_	–
RbUO_3_	t_2g_	U-*d*_*xy*_, U-*d*_*xz*_, U-*d*_*yz*_	O-*p*_*x*_
	e_g_	U-*d* 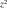 , U-*d* 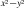	O-*p*_*y*_, O-*p*_*z*_
	A	U-*d*_*xy*_, U-*d*_*xz*_, U-*d*_*yz*_	O-*p*_*x*_
	A 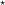	U-*d*_*xy*_, U-*d*_*xz*_, U-*d*_*yz*_	–
	A 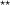	U-*d*_*xy*_, U-*d*_*xz*_, U-*d*_*yz*_	–
	B	U-*d* 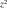 , U-*d* 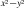	O-*p*_*y*_, O-*p*_*z*_
	C	U-*d* 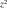 , U-*d* 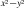	O-*p*_*x*_
