# Supplementary material for: Crystal-field splitting strength of U-6d orbitals in NaUO3, KUO3 and RbUO3
Source: J Synchrotron Radiat. 2025 Jul 30;32(Pt 5):1257–64. doi: 10.1107/S1600577525005156 (PMC12416429; doi:10.1107/S1600577525005156)
Supplement: Supplementary file 1 [file s-32-01257-sup1.pdf]

JOURNAL OF SYNCHROTRON RADIATION

Volume 32 (2025)

Supporting information for article:

Crystal-field splitting strength of U-6*d* orbitals in $NaUO_3$, KUO_3 and $RbUO_3$

Simon Orlat, Igor Prozheev, Ine Arts, Gregory Leinders, Elena Bazarkina, Kristina Kvashnina, Filip Tuomisto, Philippe Martin, Philippe Moisy and René Bes

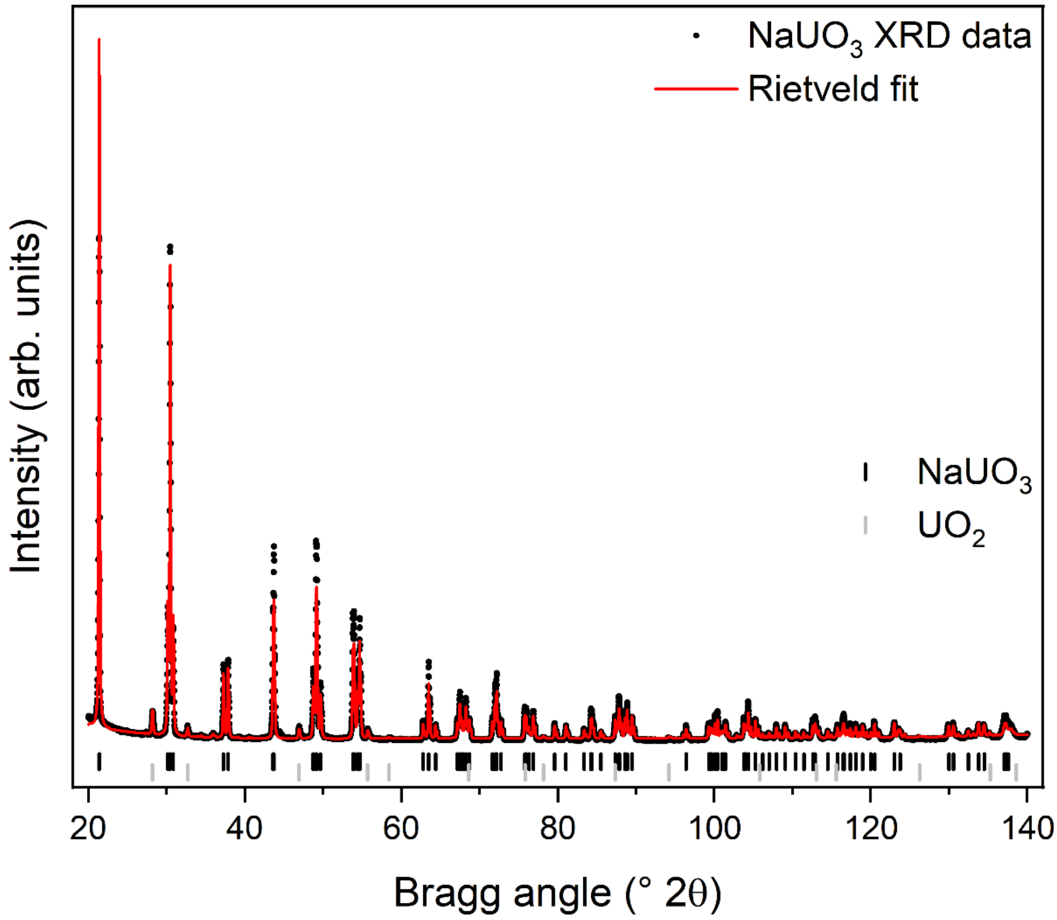

Figure S1: Diffraction pattern of the $NaUO_3$ sample (black dots) with resulting fit after Rietveld refinement (red line), χ = 7.2. Vertical lines at the bottom of the plot indicate reflections associated to $NaUO_3$ (black) and UO_2 (grey).

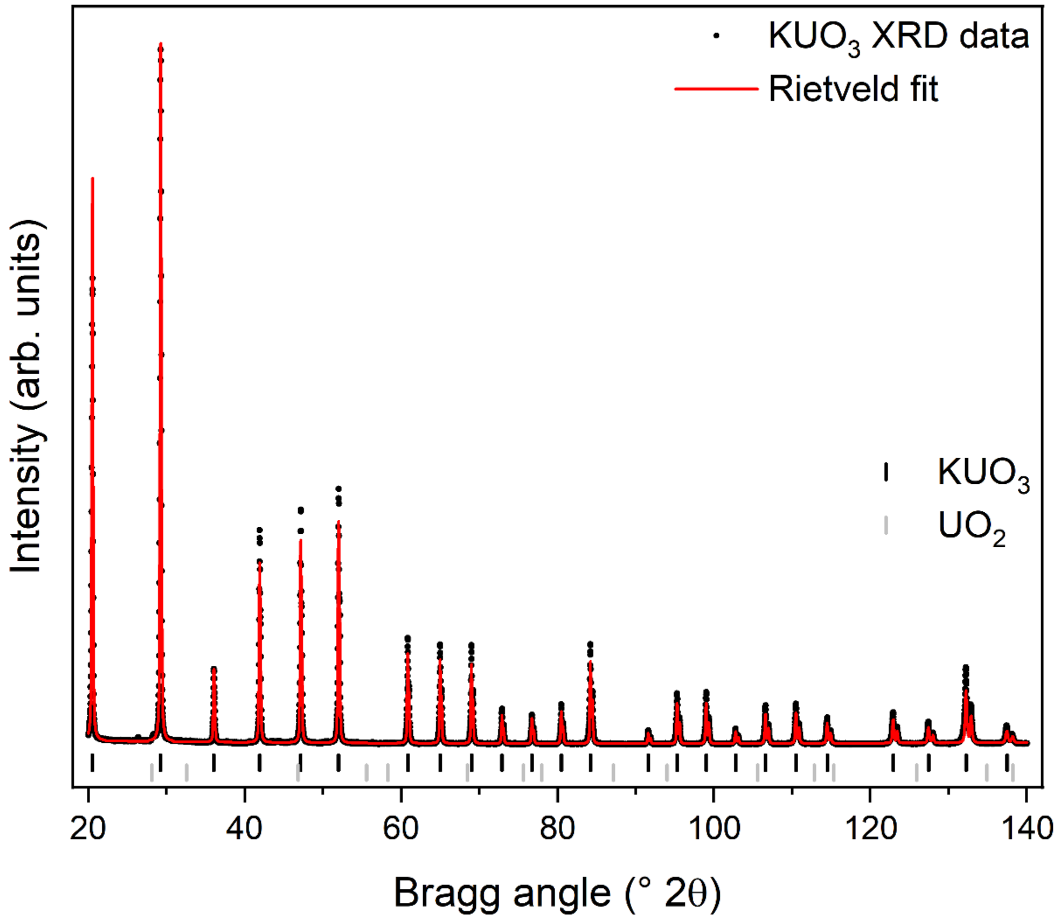

Figure S2: Diffraction pattern of the KUO_3 sample (black dots) with resulting fit after Rietveld refinement (red line), χ = 5.4. Vertical lines at the bottom of the plot indicate reflections associated to KUO_3 (black) and UO_2 (grey).

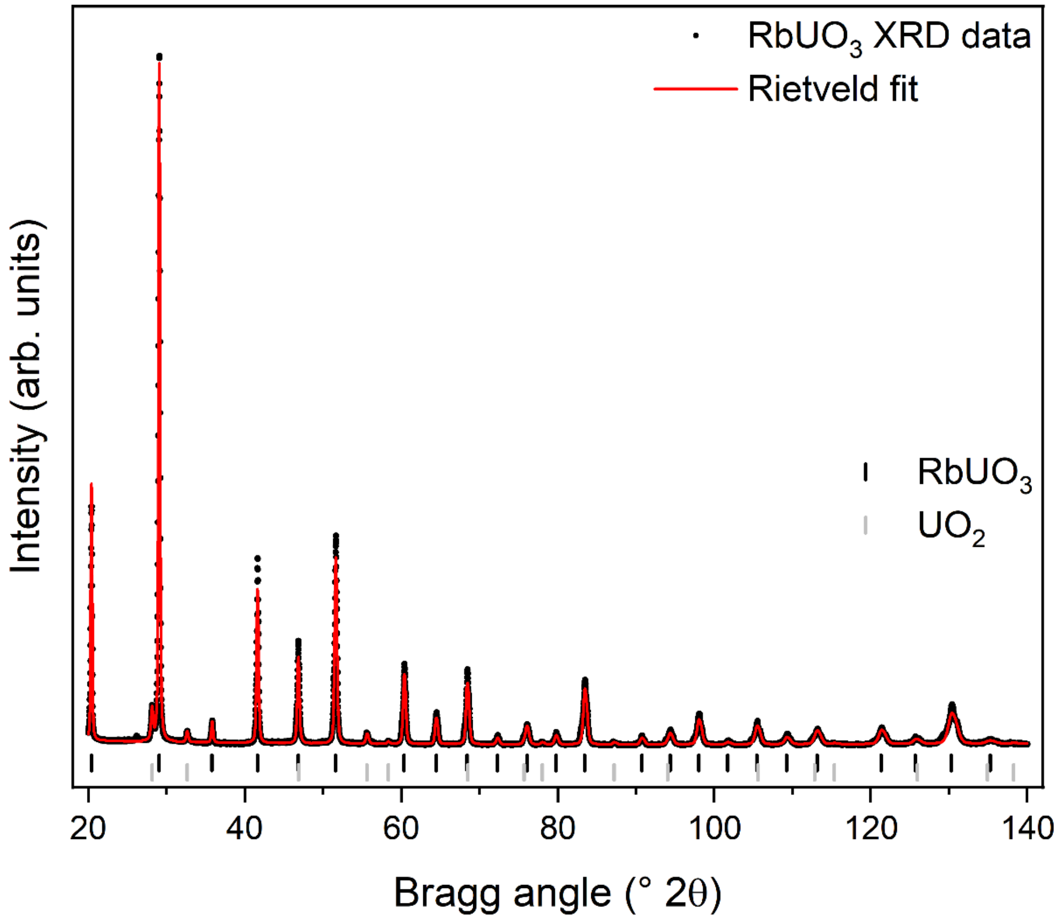

Figure S3: Diffraction pattern of the $RbUO_3$ sample (black dots) with resulting fit after Rietveld refinement (red line), χ = 3.0. Vertical lines at the bottom of the plot indicate reflections associated to $RbUO_3$ (black) and UO_2 (grey).

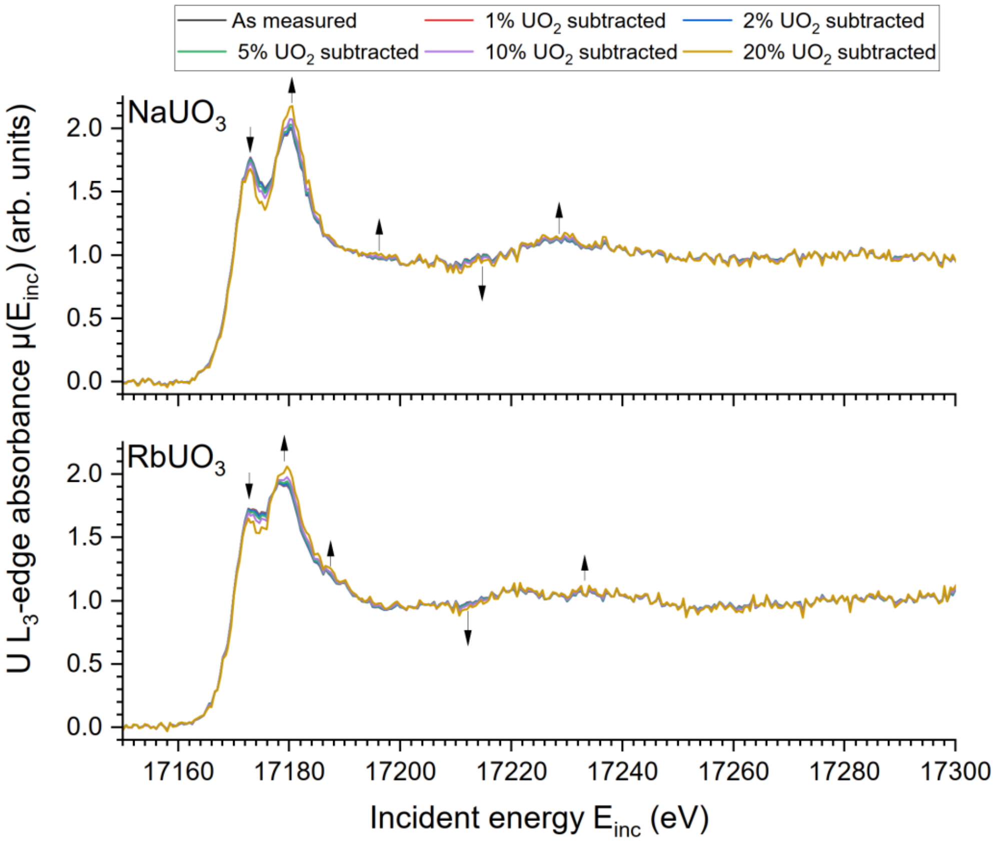

Figure S4: Qualitative analysis study of the effect of the presence of a few percentage of UO_2 as impurity on the HERFD-XANES spectral features. The experimental spectra of UO_2 was subtracted from the expeimental spectra of $NaUO_3$ and $RbUO_3$, varying the concentration of the subtracted UO2 from 1 to 20 %. Black arrows are indicating the trends in teh spectral change following the UO_2 signal subtraction. Only vertical changes are observed, no energy shift of spectral features occurs due to UO_2 as an impurity.

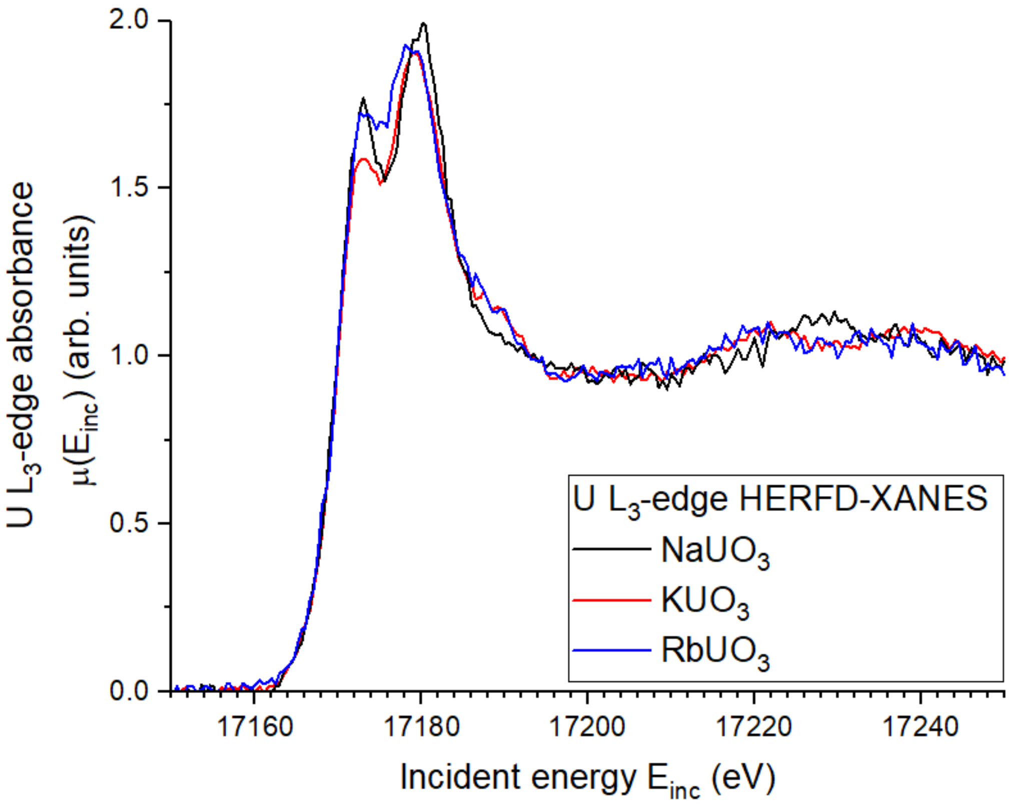

Figure S5: Superimposed U L3-edge HERFD-XANES experimental spectra for direct comparison of spectral features between NaUO3, KUO3 and RbUO3.

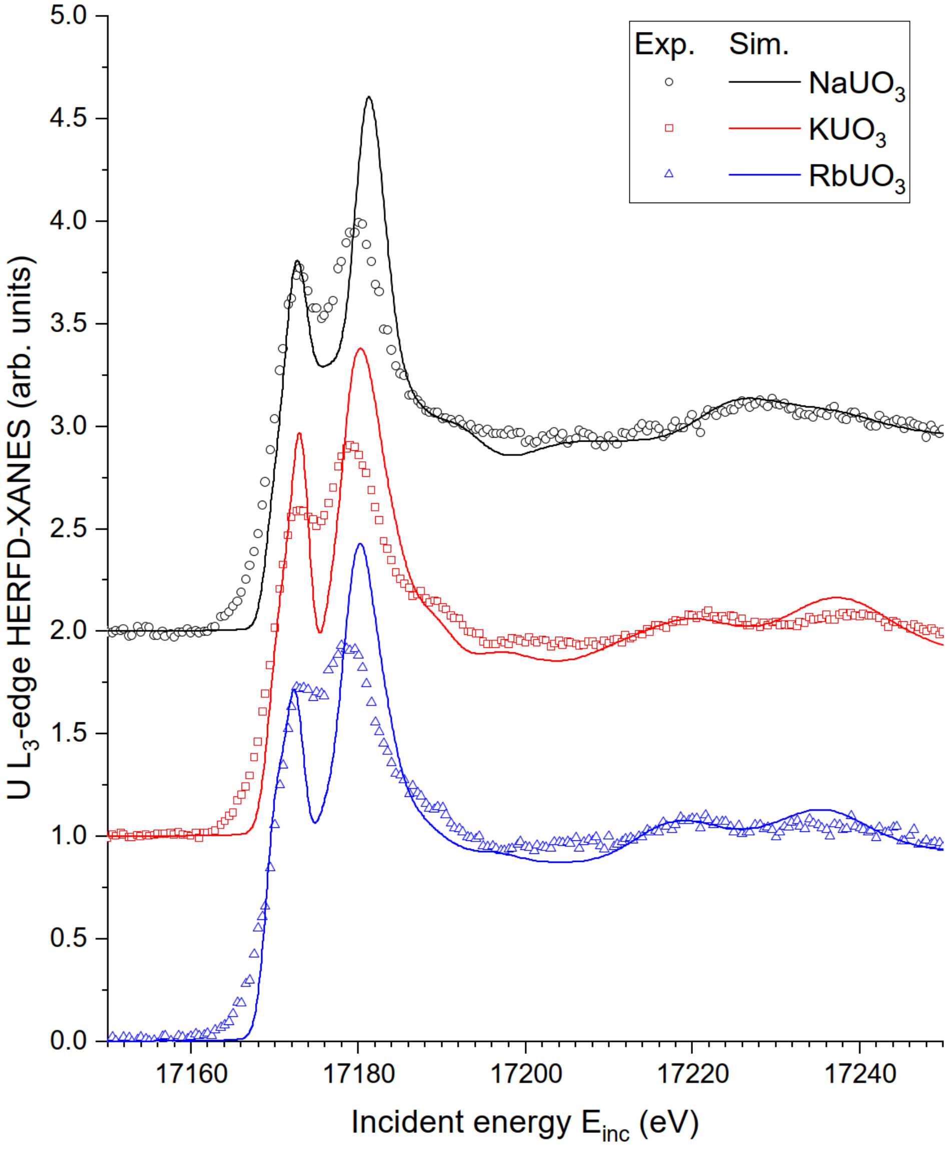

Figure S6: Comparison of experimental and simulated spectra after convolution with 2.2 eV gaussian broadening (accounting for the overall experimental resolution) and the core-hole broadening from the intermediate state, i.e. the U $O_{4,5}$ orbital, accounting for an additional Lorentzian broadening of 0.1 eV. Other convolution parameters of FDMNES were taken as default.

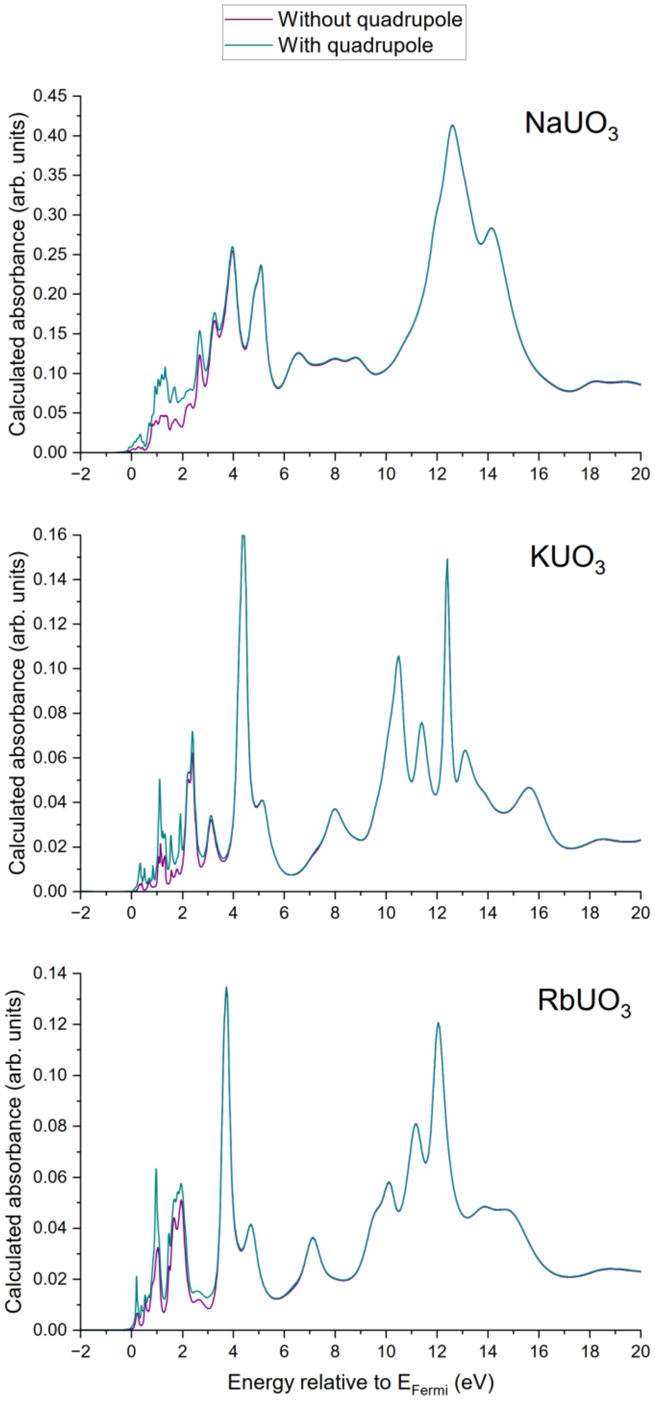

Figure S7: Comparison of the calculated spectra with and without the quadrupolar transition allowed for $NaUO_3$, KUO_3 and $RbUO_3$. The relative intensity changes are quite limited for KUO_3 and $RbUO_3$, while being slightly more pronounced for $NaUO_3$. owing a probably less pronounced hybridization of the U-5f and U-6d orbitals due to structural distortion.

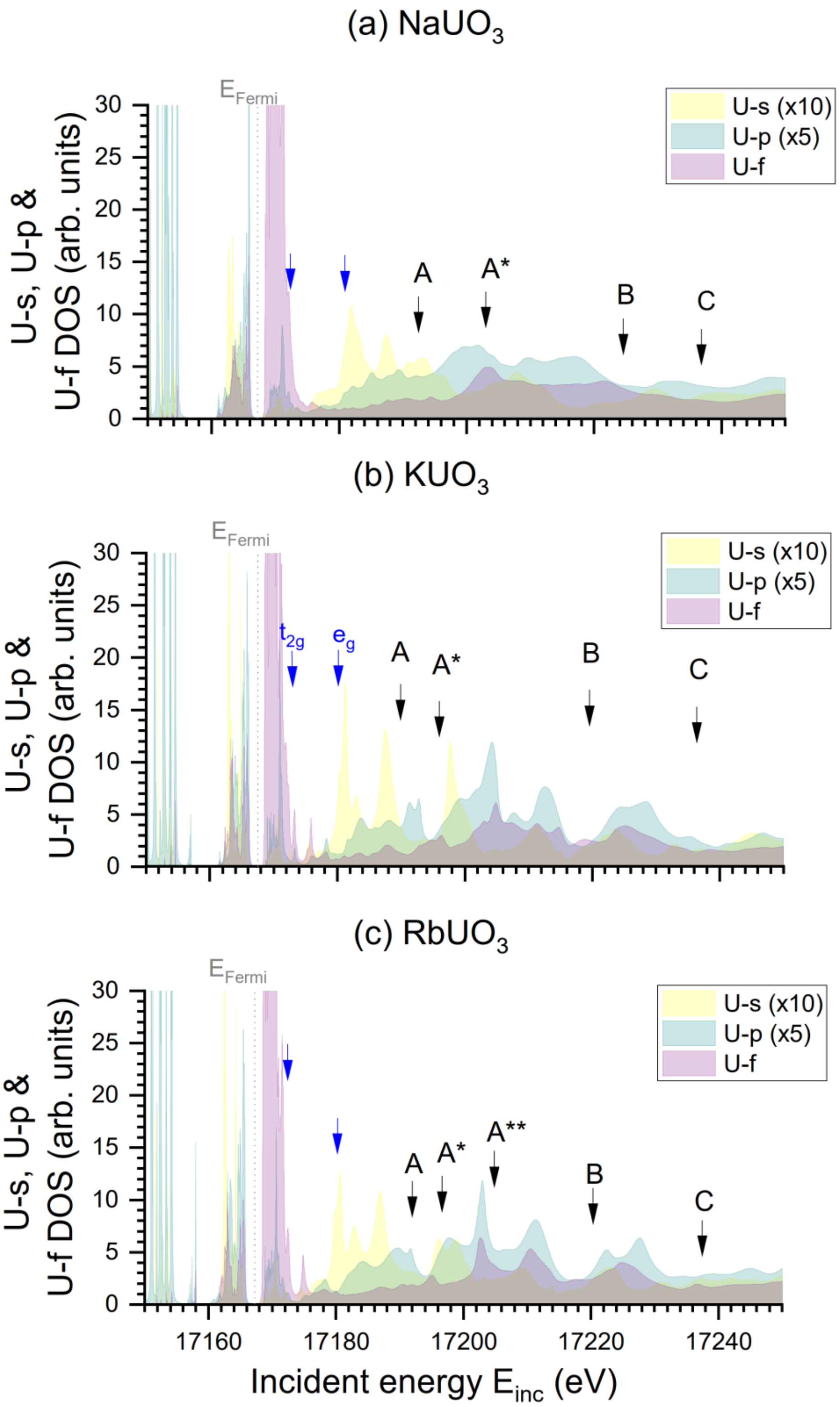

Figure S8: Calculated U-s, U-p and U-f partial DOS for (a) NaUO3, (b) KUO3 and (c) RbUO3. The Fermi energy level is indicated with a vertical dotted line. Blue arrows are pointing the crystal field splitted U-d orbitals (the so-called t2g and eg). The position of the post-edge features A, A⋆, A⋆⋆, B and C from the simulated spectra are indicated by black arrows.

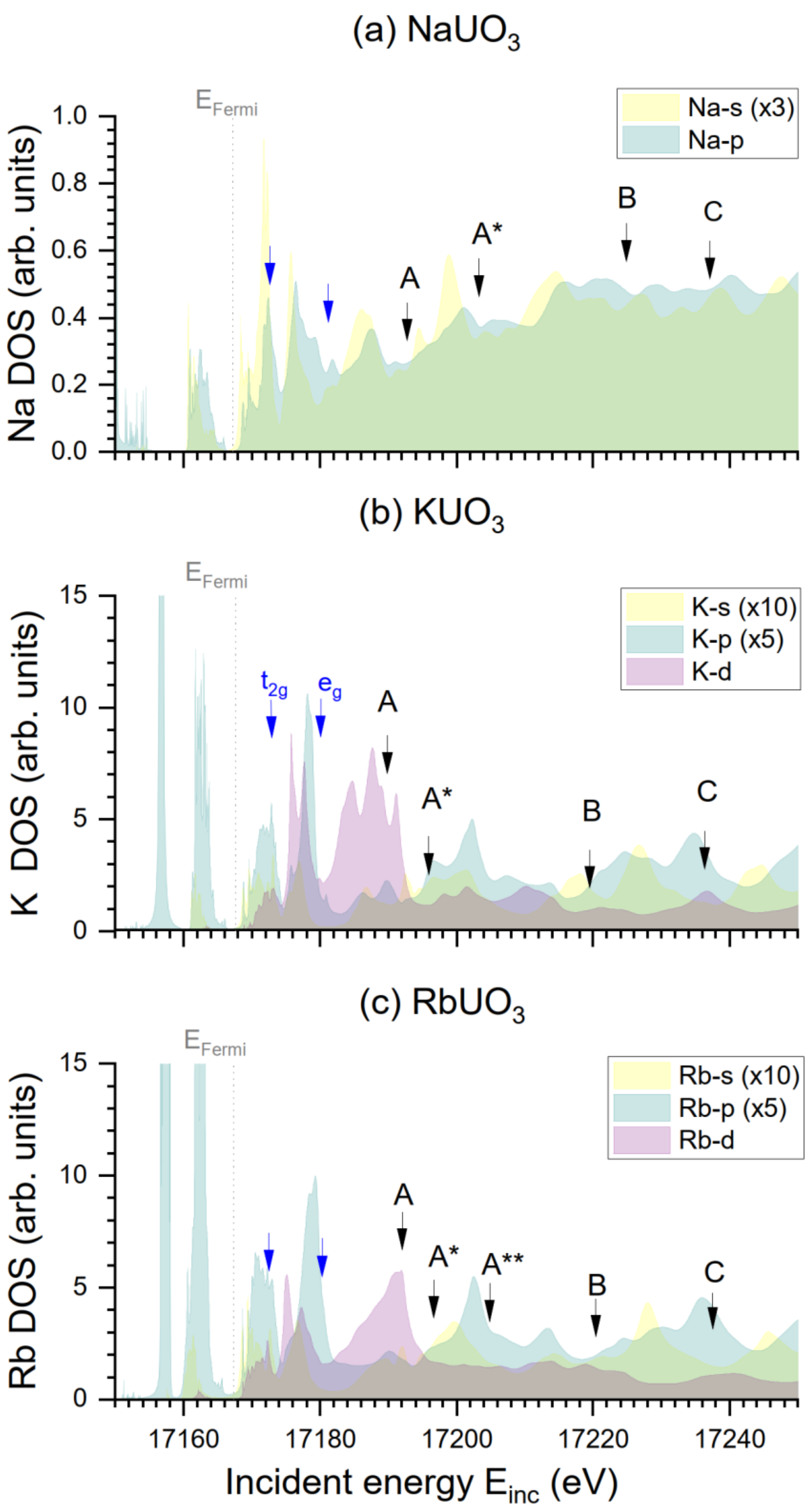

Figure S9: Calculated partial DOS of the counter ion for (a) NaUO3, (b) KUO3 and (c) RbUO3. The Fermi energy level is indicated with a vertical dotted line. Blue arrows are pointing the crystal field splitted U-d orbitals (the so-called t2g and eg). The position of the post-edge features A, A⋆, A⋆⋆, B and C from the simulated spectra are indicated by black arrows

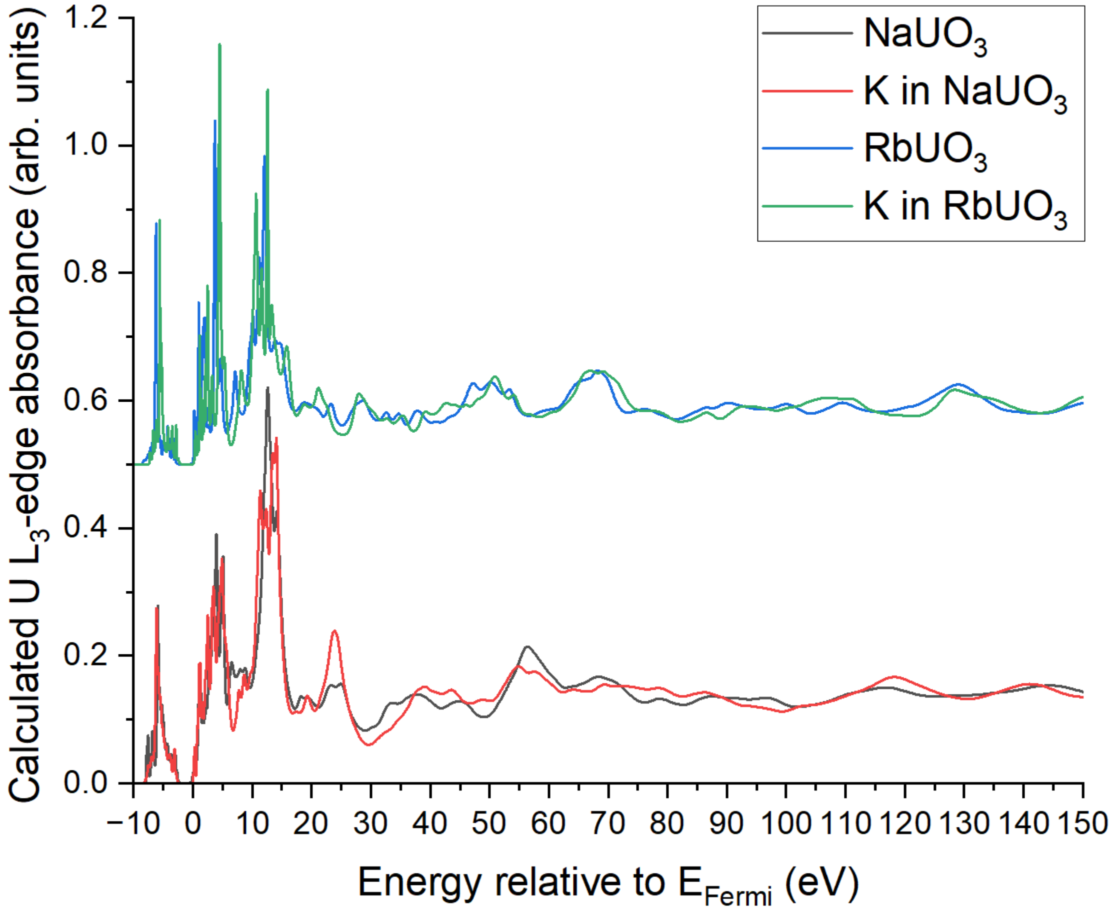

Figure S10: Study of the effect of the counter-ion through comparison of the calculated spectra of $NaUO_3$ and $RbUO_3$ with the corresponding spectra having the counter-ion replaced by K without changing the structure of the uranate.
